# Relationship between Object-Related Gestures and the Fractionated Object Knowledge System

**DOI:** 10.1155/2007/241670

**Published:** 2007-08-22

**Authors:** Angela Bartolo, Meike Daumüller, Sergio Della Sala, Georg Goldenberg

**Affiliations:** ^1^Laboratoire URECAPsychologie – Université Charles-de-Gaulle Lille3France; ^2^Neuropsychological DepartmentBogenhausen HospitalMunichGermany; ^3^Human Cognitive NeurosciencePsychology – University of EdinburghUK

**Keywords:** Limb apraxia, action semantic, mechanical problem solving, object function, brain damage, conceptual knowledge

## Abstract

The praxic semantic system comprises a conceptual knowledge system, which stores functional information about objects, and an action knowledge system, which stores information about the correct manipulation of objects. Moreover, mechanical problem solving abilities permit to take advantages from objects structure to use unfamiliar tools or discover alternative ways of using familiar tools. This study aims at investigating whether conceptual knowledge, action knowledge and mechanical problem solving abilities intervene in the production of gestures with objects (i.e. pantomimes and object use) by testing a group of brain damaged patients. Results showed that the mechanical problem solving abilities are not sufficient to produce pantomimes and that only severe deficits in the praxic semantic system would affect the production of these gestures. Furthermore, a double dissociation was observed between mechanical problem solving abilities and the capacity to use multiple objects. Overall, the results indicate that the praxic semantic system can be disrupted at different levels, suggesting that the semantic system for object has to be conceived as fractionated in different entities.

## 1. Introduction

Models of limb apraxia usually posit that object use is processed by two systems: a praxic semantic system, which refers to knowledge about objects, and a production system, which encompasses sensory motor components for executing gestures [6,17,18]. The praxic semantic system could be further dichotomised into a *conceptual knowledge* system, containing information about objects and their function, and an *action semantic* system, which comprises information about the appropriate actions to be performed with a given object [4, 5,12]. Buxbaum and Saffran [4] reported a double dissociation between conceptual knowledge and action semantic. This double dissociation has been partially confirmed by neuroimaging studies [3,13].

Goldenberg and Hagmann [7] suggested that knowledge about object use can be based also on *mechanical problem solving abilities*. These allow one to infer the object function from its structure, and permit the uncovering of alternative ways of making use of familiar tools and possible uses of unfamiliar tools (see also [16]). Therefore, the action semantic refers to the precise gestures to be executed in a functional context ("instructions to use" [7, p. 581]), while the mechanical problem solving abilities refer to actions triggered by the physical attributes of an object.

Aim of the current paper is to investigate the role of the praxic semantic system and the mechanical problem solving abilities in producing gestures with objects in a group of brain damage patients.

## 2. Methods

### 2.1. Participants

Six patients (4 males and 2 females) from the Neuropsychological Department of Bogenhausen Hospital

ISSN 0953-4180/07/$17.00 © 2007 – IOS Press and the authors. All rights reserved in Munich (Germany) were included in the study. They all had a focal brain lesion. MRI scans showed that all patients but Cases 2 and 3 presented with frontal damage (see Table 1), and only Case 6 had right brain damage. A Rule Detection task measured general intelligence [11], only two patients scored below cut-off. To exclude patients with visual agnosia, all participants were screened with a visual object matching task (eg. phone-mobile phone – 18 items). Ten volunteers matched for age and education agreed to act as controls.

### 2.2. Procedures

A series of tasks assessing the praxic semantic system, the mechanical problem solving abilities, and the production system have been included (see below). The pantomime production task, the mechanical problem solving abilities tasks, and the tasks assessing the praxic semantic systems were assessed in three different sessions. The object-manipulation knowledge task and the function knowledge task were counterbalanced across participants. In a different session the participants underwent the multiple object use task. Different objects were used in the different tasks.

#### 2.2.1. Tasks assessing the praxic semantic system

Conceptual knowledge tasks: (i) object-relation knowledge – 16 items: match the line-drawing pictures of two objects which functionally interact when used (e.g., teapot-cup) in the presence of a distracter (e.g., glass); (ii) object-function knowledge – 15 items: match the line-drawing pictures of two objects subserving the same or closely similar functions (e.g., jug – bottle) in the presence of a distracter (e.g., pot). Following Buxbaum and Saffran's procedure [4,5], the objects employed in each trial differed in their manner of manipulation.

Action knowledge tasks: (i) pantomime recognition – 21 items: match the gesture executed by the examiner (eg, to cut) to the line-drawing picture of the object (eg. knife) in the presence of three distracters (eg, iron, screwdriver, saw) and (ii) object-manipulation knowledge[1] – 16 items: match the line-drawing pictures of two objects having the same manner of manipulation (e.g. pipe-penny whistle) in the presence of a distracter (e.g., spoon). Following Buxbaum and Saffran's procedure [4,5], the objects within each trial differed in their function.

For the conceptual and action knowledge tasks, a pass/fail score (1/0) was attributed to each item.

[1]Participants were asked to avoid performing the pantomime of the objects.

#### 2.2.2. Task assessing the mechanical problem solving abilities

Novel tool test – 6 items [7]. Participants had both to choose among three novel tools the one useful to lift a cylinder (*tool selection*) and show the best use of it (*tool use*). Two points were credited for each correct choice/use on the first attempt, one point on the second attempt and zero if they failed on both attempts. The total score for both the tool selection and tool use ranged from 0 to 12. If participants failed in the tool selection, the appropriate tool was given to assess tool use.

#### 2.2.3. Tasks assessing the production system

(i) Pantomime production task – 10 items: produce pantomimes of objects whose photographs were presented [8]. Maximum score for each item was 3 and penalties were given for errors like grip, movement and distance-to-goal. Total score ranged from 0 to 30 and (ii) multiple object use tasks: a) prepare a cup of coffee and b) fix a tape recorder [9]. The action steps necessary to complete the coffee task were 21, to complete the tape recorder task were 18. For each step the scores were 0 (wrong), 1 (correct with hesitation) and 2 (flawless); maximum score for the coffee task was 42, for the tape recorder task was 36.

Individual performances were analysed in terms of multiple-single case approach. For all tests, including the visual object matching task (Table 1), cut-off scores were calculated as two standard deviations below the means achieved by the ten controls, rounded to the nearest full digit minus one additional point (to be conservative).

## 3. Results

### 3.1. Pantomimes

Cases 1 and 3 scored below cut-off in the object-function and -manipulation knowledge tasks and in the mechanical problem solving abilities task; in addition they failed in the production of pantomimes (Table 2).

Case 5 scored below cut-off in the object-manipulation knowledge task (9/16), but performed near ceiling in the production of pantomimes (28/30). Case 2 also was impaired in the object-manipulation knowledge task (4/16), but he failed the production of pantomimes task (16/30).

Both Case 2 and 5 scored above cut-off in the object-function knowledge task (13/15 and 12/15 respective-

Table 1
Demographical and clinical data of the patients included in the study

| Cases | Gender | Age | Education (years) | Days from onset | Lesion side | Site of lesion | Rule detection task[1] | Visual-object matching task[2] |
|---|---|---|---|---|---|---|---|---|
| 1 | Male | 56 | 13 | 360 | L | F+T | 108 | 18 |
| 2 | Male | 54 | 14 | 840 | L | T+P | 82* | 18 |
| 3 | Female | 67 | 14 | 150 | L | T | 91 | 17 |
| 4 | Male | 54 | 16 | 60 | L | F+T | 100 | 18 |
| 5 | Female | 40 | 11 | 60 | L | F+T+P | 88 | 18 |
| 6 | Male | 58 | 15 | 270 | R | F+T | 73* | 17 |

F = frontal, L = left, P = parietal, R = right, T = temporal.
[1]mean = 100, cut-off = 85.
[2]Controls' score: range 16–18, mean = 17.6, SD = 0.7, cut-off: 15.
*Pathological score.

Table 2
Scores achieved by six brain damage patients entering the study in tasks assessing praxic semantic, mechanical problem solving abilities, production of pantomimes, and use of multiple objects. Scores below cut-off are marked by asterisks

| | | Praxic semantic system | | | | Mechanical knowledge | | Production system | | |
|---|---|---|---|---|---|---|---|---|---|---|
| | | Conceptual knowledge | | Action knowledge | | Novel Tool Task | | Pantomime (0–30) | Multiple object use tasks | |
| | | Object-relation (0–16) | Object-function (0–15) | Pantomime recognition (0–21) | Object-manipulation (0–16) | Selection (0–12) | Use (1–12) | | Coffee (0–42) | Tape Recorder (0–36) |
| Normal | Min-max | 15–16 | 14–15 | 19–21 | 13–16 | 12 | 11–12 | 26–30 | 35–42 | 28–35 |
| Controls | Means (SD) | 15.8 (0.4) | 14.4 (0.5) | 20.4 (0.8) | 14.1 (0.9) | 12 (0) | 11.9 (0.2) | 27.7 (1.2) | 39.1 (2.3) | 32.2 (3.3) |
| | Cut offs | 14 | 12 | 18 | 11 | 11 | 11 | 24 | 34 | 25 |
| | Cases | | | | | | | | | |
| | 1 | 15 | 10* | 18 | 8* | 7* | 9* | 13* | 36 | 28 |
| | 2 | 15 | 13 | 19 | 4* | 10* | 10* | 16* | 32* | 29 |
| | 3 | 14 | 9* | 19 | 7* | 4* | 10* | 7* | 34 | 18* |
| | 4 | 11* | 11* | 15* | 4* | 12 | 12 | 2* | 21* | 23* |
| | 5 | 16 | 12 | 19 | 9* | 11 | 12 | 28 | 39 | 28 |
| | 6 | 16 | 14 | 21 | 14 | 9* | 12 | 26 | – | – |

ly). Yet, Case 2 was impaired in the selection (10/12) and use (10/12) of novel tools, whereas Case 5 performed above cut-off in the mechanical problem solving abilities tasks (11/12 selection and 12/12 use of novel tools). Taken together, these two cases suggest a possible role of the mechanical problem solving abilities in producing gestures. However, Case 6 who scored above cut-off in all tasks assessing conceptual and action knowledge (see Table 2), and was above cut-off in the production of pantomimes (26/30), was instead impaired in the mechanical problem solving abilities task (9/12). In contrast, Case 4 who scored below cut-off in all tasks taxing conceptual and action knowledge, performed at ceiling in the mechanical problem solving abilities task but markedly failed the production of pantomimes (2/30).

### 3.2. *Multiple object use*

Case 4 was impaired in both tasks assessing multiple objects use (21/42 coffee and 23/36 tape recorder), though he scored at ceiling in the mechanical problem solving abilities task, whereas Case 1 performed above cut-off in the coffee (36/42) and tape recorder (28/36) tasks, yet showing a clear deficit in the mechanical problem solving abilities task (7/12 selection and 9/12 use of novel tools). Interestingly, they were both impaired in the object-function and -manipulation knowledge tasks (Table 2).

## 4. Discussion

Goldenberg and Hagmann [7] proposed that action semantic knowledge could be explored by means of a production of pantomimes task. Yet it is difficult to distinguish a semantic from a motor deficit if patients failed in this task. In this study, we have included an object-manipulation knowledge task that permits to circumvent the motor components embedded in the production of pantomimes. Indeed, in the objectmanipulation task participants had to recall the correct gesture compatible with the object without actually producing the pantomime.

Five out of six patients failed the object-manipulation knowledge task and they were also impaired in the production of pantomimes. Interestingly, Case 5 scored below cut-off in the object-manipulation task, but was close to ceiling in performing pantomimes. A possible explanation is that she was able to integrate her spared semantic (object-function) knowledge and her knowledge about objects use in order to produce the correct pantomimes (see Bartolo et al. [1]). Alternatively she could have capitalised on her spared mechanical problem solving abilities to infer the correct pantomime (see Hodges et al. [10]). This latter interpretation however is not supported by the profile of two other cases. Case 4 who was at ceiling in the mechanical problem solving abilities task but failed conceptual and action semantic knowledge tasks, scored very poorly in the production of pantomimes. In contrast, Case 6 who had impaired mechanical problem solving skills coupled with intact object-manipulation knowledge was able to produce pantomimes. Taken together, these three cases suggest that one needs not rely on mechanical problem solving abilities to produce gestures. In particular, it is worth noticing that Case 4 had some recollection of the objects. Indeed, his ability to match two versions of the same object was spared (see Table 1). It follows that, as it happens for the imitation of gestures [2,6] and the reproduction of words [14,15], once an objects is recognised, the lexical route is automatically activated, even if it is damaged, reducing the possibility to switch to alternatives routes for producing gestures (Case 4). Based on their spared abilities, patients could rely either on the action semantic (Case 6) or on conceptual knowledge (Case 5) to produce pantomimes.

The outcome of this study suggests that the mechanical problem solving abilities are not sufficient to deduce gestures to be performed with objects and that only severe deficits in the praxic semantic system would dramatically affect patients' performance on this task (as in Case 4). The double dissociation between mechanical problem solving abilities and multiple objects use tasks (Cases 1 and 4) is in line with the lack of correlation between these two tasks reported by Hartmann et al. [9]. It follows that inference of an object function from its structure plays only a marginal role in everyday multi-objects sequencing tasks.

In sum, the praxic semantic system is better thought of as a fractionated entity that encompasses separate functions dealing with conceptual and action knowledge, which in turn are distinct from mechanical problem solving abilities.

## Acknowledgments

We thank Dr Karoline Hartmann for helping with the testing sessions. Special thanks to all patients who participated in the study.
